# Workplace Social Capital, Professional Identity, and Work‐Related Quality of Life Among Nurses: A Latent Profile Analysis

**DOI:** 10.1111/inr.70192

**Published:** 2026-06-22

**Authors:** Wenjuan Zhu, Xiumei Wang, Li Zhang, Dinuo Xin, Xiaoxia Chen, Na Xu, Ying Wang, Wanling Li

**Affiliations:** ^1^ Nursing Department Shanxi Bethune Hospital Shanxi Academy of Medical Sciences Third Hospital of Shanxi Medical University Tongji Shanxi Hospital Taiyuan China; ^2^ Nursing Department Yuncheng Central Hospital Yuncheng Shanxi China; ^3^ Nursing Department Tongji Hospital Tongji Medical College Huazhong University of Science and Technology Wuhan Hubei China; ^4^ Comprehensive Medical Department Tongji Hospital Tongji Medical College Huazhong University of Science and Technology Wuhan Hubei China

**Keywords:** Latent profile analysis, nurses, professional identity, psychosocial heterogeneity, turnover intention, workplace social capital, work‐related quality of life

## Abstract

**Aim:**

To identify latent organizational–psychosocial profiles among clinical nurses based on their perceptions of workplace social capital (WSC), professional identity (PI), and work‐related quality of life (WRQoL), and to explore demographic and occupational predictors of profile membership.

**Background:**

WSC, PI, and WRQoL are critical factors influencing nurses’ occupational well‐being, job satisfaction, and retention. With increasing managerial focus on these elements, the potential for these factors to form distinct latent profiles remains to be explored.

**Methods:**

A cross‐sectional survey was conducted among 1,630 nurses from three hospitals in China. Latent profile analysis identified subgroups based on 14 dimensions across the WSC, PI, and WRQoL scales. Multinomial logistic regression was used to examine factors associated with subgroup membership.

**Results:**

Three latent profiles were identified: Resource‐Deprived and High‐Stress Group, Adaptive–Stable Group, and High Resource–High Identity Group. Multinomial logistic regression showed that lower hierarchical (N2), lower monthly income (≤5000 RMB), specific department assignments (outpatient and medical technology), and strong turnover intentions were significantly associated with membership in the Resource‐Deprived and High‐Stress Group. Nurses in this group were also more likely to experience higher stress and resource deprivation compared with those in the other profiles.

**Conclusion:**

Nurses display significant organizational–psychosocial heterogeneity, influenced by structural factors such as hierarchical position, clinical specialty, income level, and turnover intention.

**Implications for Nursing:**

Nurse managers should recognize the heterogeneity within nursing groups and, for those in resource‐deprived and high‐stress profiles, optimize shift schedules, reduce workloads, and provide psychological support to alleviate physical and emotional stress.

**Implications for Nursing Policy:**

Policymakers should design tailored professional development initiatives for different nurse subgroups, ensure equitable and competitive remuneration systems, and enhance nurse well‐being and retention.

## 1 Introduction

Nurse shortages have emerged as a critical global public health concern. According to the 2025 report released by the International Council of Nurses (2025), the nursing workforce is facing unprecedented physical and psychological health challenges: 23%–61% of nurses report symptoms of anxiety or depression, 18% experience burnout, and 61.7% of countries have reported a significant increase in nursing workloads. Meanwhile, as population aging accelerates and the burden of chronic diseases continues to grow, the demand for nursing services is expanding rapidly. However, salary growth and professional recognition have failed to keep pace, undermining the stability and attractiveness of the profession. In 48.4% of countries, nurse turnover has intensified (International Council of Nurses 2025). Nurses’ organizational–social–psychological environment, including workplace support, professional identity (PI), and work‐related quality of life (WRQoL), directly influences how nurses perceive their roles within the healthcare system. Accordingly, nursing managers have increasingly focused on workplace support, PI, and nurses’ WRQoL, as these factors are closely linked to well‐being, job satisfaction, and retention (Pressley and Garside 2023; Xu et al. 2024). However, existing studies have mainly examined their separate effects or interrelationships, while little is known about whether they cluster into distinct latent profiles among nurses (Babapour et al. 2022 **;** Li et al. 2024). Identifying different nurse subgroups may therefore inform targeted strategies to support nurse well‐being and retention, thereby ensuring care quality and advancing universal health coverage.

Work‐related quality of life (WRQoL) is a multidimensional concept that reflects employees’ subjective perceptions and evaluations of their work environment, organizational support, career development opportunities, and work–life balance (Van Laar et al. 2007; Silarova et al. 2022). WRQoL influences workplace social capital (WSC) and PI: higher WRQoL is linked with greater professional engagement, job satisfaction, and a stronger PI (Melnyk et al. 2018; Rashid and Amin 2024). Nurses’ perceptions of WRQoL are shaped by factors such as workload, work stress, and organizational support, which can also impact their PI. For instance, a negative work environment may reduce PI and job satisfaction (Akter et al. 2019). Studies have shown that factors such as more work experience, married status, and working in specialty units are associated with higher WRQoL among nurses (Al Mutair et al. 2022). These factors contribute to greater job satisfaction and better overall work experiences. Therefore, understanding WRQoL is essential for improving job satisfaction, retention, and overall well‐being among nurses.

WSC refers to the network of workplace relationships based on mutual understanding, trust, reciprocity, and team cohesion among organizational members (Xu and Stark, 2021). WSC enhances nurses’ job satisfaction, alleviates work‐related stress, and reduces turnover intentions. Moreover, strong WSC can improve both WRQoL and PI, providing a supportive environment for nurses to thrive (Kida et al. 2023; Xu et al. 2021). Studies suggest that nurses’ perceptions of WSC vary across individuals and are influenced by factors such as education level, work experience, and job position (Pittman et al. 2019; Firouzbakht et al. 2018). In the increasingly complex healthcare environment, WSC serves as a critical resource supporting nurses’ professional development, team stability, and organizational resilience.

PI denotes an individual's perception of their professional role, emotional affiliation, and value recognition, forming a core part of their vocational self‐concept (Fitzgerald 2020). PI encompasses not only professional knowledge and skills but also ethics, values, and the integration of personal beliefs with societal expectations (Johnson et al. 2012). Contemporary healthcare environments present challenges to PI, particularly when nurses are viewed predominantly as “compassionate caregivers.” This stereotype may inadvertently undermine nurses’ professional image, which requires independent judgment and advanced technical expertise (Andrew 2012). This discrepancy can weaken PI, as nurses may feel undervalued. These feelings are often compounded by the quality of WSC, where a lack of support, trust, and respect can exacerbate role ambiguity, diminishing PI. Front‐line nurses, in particular, may experience this challenge more acutely due to the perceived “invisibility” of their contributions (Landis et al. 2020). A comprehensive review of nurses’ PI indicates that demographic variables, such as age, gender, work experience, and education, interact with other factors to significantly influence the development and strength of nurses’ PI, highlighting the importance of considering these variables when studying PI in nursing populations (Mao et al. 2021).

Occupational health psychology (Quick and Tetrick 1993) provides a framework for understanding how work conditions impact employees’ psychological and physical well‐being. WRQoL, which reflects employees' overall work experience—including work environment, stress, and work–life balance—can enhance PI by fostering job satisfaction and personal achievement. In this context, nurses’ WRQoL is influenced not only by their work environment but also by the social resources available to them through workplace relationships. Social capital theory (Liukkonen et al. 2004) highlights the role of social relationships, trust, and community support in the workplace. WSC, based on mutual trust and respect, plays a critical role in shaping psychological well‐being. In nursing, WSC enhances both WRQoL and PI by helping nurses cope with stress and job demands. Thus, we hypothesize that WSC positively influences nurses’ WRQoL and PI. The enhancement of these constructs is theoretically linked to broader professional benefits, including improved work outcomes and greater engagement.

## 2 Aim of the Study

This study harnessed principles from occupational health psychology and social capital theory to better understand the inherent heterogeneity among nursing staff across organizational and psychological dimensions. Using latent profile analysis (LPA), we sought to identify potential subgroups of nurses based on their combined levels of WSC, PI, and WRQoL, and to explore their organizational–psychosocial profiles. Furthermore, the present study analyzed subgroup characteristics and influencing factors to support the precise identification of at‐risk populations and the development of tailored management strategies aimed at improving nurse well‐being and fostering sustainable workforce development. The specific objectives were to (1) identify latent subgroups of nurses based on WSC, PI, and WRQoL profiles; (2) examine the distribution of demographic and work‐related characteristics across subgroups; and (3) determine independent predictors of membership in the least favorable latent profile using multivariable logistic regression.

## 3 Methods

### 3.1 Study Design and Participants

Adhering to the Strengthening the Reporting of Observational Studies in Epidemiology (STROBE) guidelines, this investigation was designed as a cross‐sectional, descriptive, and exploratory study (**von Elm et al**. 2007). Data were collected between 10 January and 20 March 2024 across three hospitals in China—a tertiary hospital and a secondary hospital in Shanxi Province, and a tertiary hospital in Wuhan, Hubei Province—using a convenience sampling approach, with all eligible nurses invited to participate voluntarily. Notably, data from this study were harnessed in another study focusing on the mediating mechanisms among the variables; the two studies differ in aims and statistical methods and are under independent review. After obtaining approval from the directors of the nursing departments, the research team organized online briefings with head nurses across departments to provide standardized instructions regarding the study purpose and questionnaire completion. The electronic questionnaire was then distributed by head nurses via workplace WeChat groups using the Wenjuanxing platform (WJX, https://www.wjx.cn/), which allowed one submission per IP address. The questionnaire included an introductory section outlining the study aims, instructions for completion, and participants’ rights, clearly stating that participation was voluntary and that respondents could withdraw at any point. All responses were anonymous and used solely for research purposes.

### 3.2 Inclusion and Exclusion Criteria

Inclusion criteria were: (1) possession of a valid Nurse Practitioner License issued by the People's Republic of China; (2) currently engaged in front‐line clinical nursing work with a minimum of one year of continuous clinical experience; (3) ability to independently understand the questionnaire and complete it electronically; (4) voluntary participation with informed consent, accompanied by submission of a fully completed questionnaire. Exclusion criteria were: (1) nursing interns, rotating nurses, or visiting nurses; (2) nursing assistants or other personnel without a registered nursing license; (3) individuals on medical, maternity, or personal leave for ≥1 month during the study period.

### 3.3 Sample Size

The sample size was estimated a priori using G*Power 3.1, based on a medium effect size (*f* ^2^ = 0.15), a significance level of 0.05, and a statistical power of 0.80. The minimum required sample size was 207 participants.Of the 1,787 questionnaires initially collected, 157 were systematically excluded based on predefined quality control criteria. Specifically, responses were removed due to completion times under 120 seconds (*n* = 127), logical inconsistencies (e.g., age < 20 or > 60) (*n* = 9), and uniform responses across all items (*n* = 11). After exclusions, 1,630 valid questionnaires were retained for final analysis, yielding an effective response rate of 91.2%. Given the requirements of LPA, a final sample of 1,630 participants was considered sufficient to ensure adequate power for subgroup identification and robust regression analyses (Spurk et al. 2020).

### 3.4 Instruments

#### 3.4.1 General Information Questionnaire

A self‐developed demographic questionnaire was used to collect participants’ general information. Variables included age, gender, marital status, number of children, educational level, professional title, clinical rank, clinical unit, years of work experience, specialty nurse qualification, average daily overtime hours, average weekly night shifts, departmental system, monthly income, and turnover intention.

#### 3.4.2 WRQoL Scale

The Chinese version of the Work‐Related Quality of Life Scale (WRQoL‐2) was used to assess nurses’ perceived quality of work life. The original scale was developed by Van Laar et al. (2007), and later culturally adapted for Chinese healthcare settings by Li et al. (2022). The Chinese version's Cronbach's α coefficient was 0.91. The WRQoL‐2 contains 33 items across seven dimensions: working conditions, work stress, control at work, work–family balance, work evaluation, general well‐being, and career satisfaction. Each item is rated on a 5‐point Likert scale ranging from 1 (“strongly disagree”) to 5 (“strongly agree”), producing a total score between 33 and 165. Higher scores indicate better perceived WRQoL. In the present study, the Cronbach's α was 0.77.

#### 3.4.3 Workplace Social Capital Scale

The Workplace Social Capital Scale, developed by Kouvonen et al. (2006), was used to assess the level of social capital in workplace settings. The Chinese version was adapted and validated by Zhang Nan et al. (2014) for healthcare professionals. The Chinese version's Cronbach's α coefficient was 0.898. It consists of eight items grouped into two dimensions: bonding and non‐bonding social capital. Each item is rated on a 5‐point Likert scale, with higher scores indicating stronger perceived workplace social capital. In the present study, the Cronbach's α was 0.93.

#### 3.4.4 Professional Identity Scale

The Professional Identity Scale for Nurses, developed by Liu (2009), was used to assess nurses’ PI. The scale consists of 30 items across five dimensions: professional cognitive evaluation, professional social skills, professional social support, professional frustration coping, and professional self‐reflection. Items are rated on a 5‐point Likert scale from 1 (“strongly disagree”) to 5 (“strongly agree”), yielding a total score between 30 and 150. Total scores are classified into three levels: low (30–90), moderate (91–120), and high (121–150). The Chinese version of the scale demonstrated a Cronbach's α coefficient of 0.938. In the present study, the Cronbach's α was 0.81.

### 3.5 Quality Control

To minimize potential bias, nurses from various departments of three hospitals were invited to participate, ensuring diversity and representativeness. Before data collection, the research team conducted online training for research assistants and designated coordinators at each participating hospital. The purpose of the training was to standardize the procedures for questionnaire distribution, informed consent communication, and data management, and it did not provide any guidance on how to answer the questionnaire. The survey was administered electronically to avoid manual data entry errors, ensure anonymity, and decrease social desirability bias.

### 3.6 Ethical Considerations

This study was reviewed and approved by the ethics committee of Shanxi Bethune Hospital, China (Approval No. YXLL‐2023‐229). Participation was entirely voluntary and anonymous. Submission of the completed online questionnaire was regarded as provision of informed consent. Participants were informed that they could withdraw from the study at any stage without penalty. All data were kept confidential, and no individual responses were accessible to nursing management or any third parties.

### 3.7 Data Analysis

LPA was conducted in Mplus 8.3, with the analysis applied to 14 dimensions across three scales—WSC, PI, and WRQoL. Model fit was evaluated using Akaike information criterion (AIC), Bayesian information criterion (BIC), and sample‐size adjusted BIC (aBIC) (lower values indicating better fit), as well as the Lo–Mendell–Rubin (LMR) and bootstrap likelihood ratio tests (BLRT). An entropy value exceeding 0.80 was generally considered indicative of good classification accuracy (Spurk et al. 2020). To assess the potential impact of common method variance (CMV) on the results, we performed Harman's single‐factor test and confirmatory factor analysis (Podsakoff et al. 2003). The results indicated that CMV was not a significant issue in the data (see Supplementary Table S3 for detailed CMV results).

Statistical analyses were performed using SPSS 23.0. Categorical variables were summarized as frequencies and percentages and compared using χ^2^ tests. Pearson correlations were used to assess associations among the main study variables. Multinomial logistic regression was used to identify factors associated with membership in the three latent profiles. Variables demonstrating significance in univariate tests were entered into the regression model, and multicollinearity was assessed using the variance inflation factor (VIF) (>5 as the cutoff). All tests were two‐tailed, with significance set at *p* < 0.05.

## 4 Results

### 4.1 Scale Reliability and Intercorrelations

The overall Cronbach's α for all survey instruments was 0.95, indicating excellent internal consistency. Reliability coefficients for each subscale are presented in Supplementary Table S1. Pearson correlation analysis revealed that the work stress dimension of the WRQoL scale was negatively correlated with all other dimensions, whereas positive correlations were found among the remaining variables (Supplementary Table S2).

### 4.2 Latent Organizational–Psychosocial Profiles Among Nurses

LPA was conducted based on the mean scores of 14 subdimensions from the WSC, PI, and WRQoL scales. Models ranging from 2 to 5 classes were sequentially estimated (Table 1). As the number of latent classes increased, the AIC, BIC, and adjusted BIC values decreased, and all models demonstrated high classification accuracy (entropy > 0.96). The three‐class model exhibited a substantial improvement in model fit (AIC = 35,049.326; BIC = 35,362.313; aBIC = 35,178.057; entropy = 0.969), with significant results for both the LMR likelihood ratio test and the BLRT (*p* < 0.001). Although the four‐class model achieved the best statistical fit (entropy = 0.978), one of its classes accounted for only 6% of the sample, limiting its theoretical interpretability and practical intervention value. Therefore, considering model fit, classification clarity, and feasibility of application, the three‐class model was selected as the optimal solution for this study.

**TABLE 1:** Model fit indices for latent profile solutions(*n* = 1,630).

Models	AIC	BIC	aBIC	Entropy	P_LMR_	P_BLRT_	Category probability
2	41,031.678	41,263.721	41,127.117	0.969	0.000	0	0.59/0.41
3	35,049.326	35,362.313	35,178.057	0.969	0.0007	0	0.42/0.37/0.21
4	30,487.128	30,881.06	30,649.152	0.978	0.001	0	0.06/0.30/0.44/0.20
5	28,603.338	29,078.215	28,798.655	0.968	0.0249	0	0.06/0.14/0.35/0.25/0.20

*Note*: ABIC, adjusted Bayesian information criterion; AIC, Akaike information criterion; BIC, Bayesian information criterion; BLRT, bootstrapped likelihood ratio test; LMR, Lo–Mendell–Rubin likelihood ratio test.

### 4.3 Labeling and Interpretation of Latent Profiles

**FIGURE 1:**
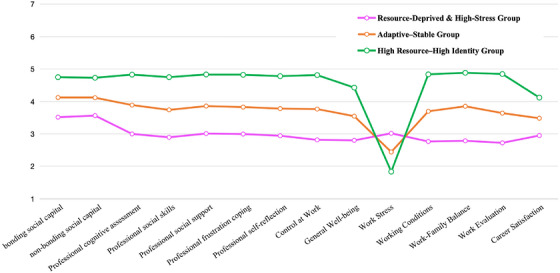
Mean scores on organizational–psychological indicators by latent nurse profile.

To visualize the distinct characteristics of each latent class, a profile plot was generated (Figure **1**). The x‐axis represents the 14 subdimensions of WSC, PI, and WRQoL, while the y‐axis reflects the mean score of each latent group on these dimensions. The results revealed the following patterns: Class 1(C1), denoted the Resource‐Deprived and High‐Stress Group (42.0%), demonstrated the lowest levels of job control, organizational support, and job satisfaction, coupled with the highest reported work stress. Class 2(C2), labeled the Adaptive–Stable Group (37.0%), exhibited moderate‐to‐high scores across most dimensions, with balanced levels of PI, work–family balance, and job satisfaction, and relatively manageable stress levels. Finally, Class 3(C3), identified as the High Resource–High Identity Group (21.0%), achieved the highest scores across nearly all dimensions, especially in organizational resources, PI, and job satisfaction, while reporting the lowest levels of stress.

### 4.4 Differences in Latent Profiles by Demographic and Occupational Factors

Univariate analyses were performed to examine differences in demographic and work‐related characteristics across the three latent classes. Significant differences were found in marital status, professional title, hierarchical position, years of clinical experience, monthly income, daily overtime duration, weekly night shifts, clinical specialty, and turnover intention (all *p* < 0.05; Table 2).

**TABLE 2:** Distribution of latent organizational–psychosocial profiles by demographic characteristics.

Variable		*N*	Class 1 (*n*, %)	Class 2 (*n*, %)	Class 3 (*n*, %)	χ^2^	*P*
Gender	Male	1,517	637(42.0)	556(36.7)	324(21.4)	3.114	0.211
	Female	113	55(48.7)	41(36.3)	17(15.0)		
Marital status	Unmarried	469	187(39.9)	197(42.0)	85(18.1)	8.696	**0.013**
	Married	1,161	505(43.5)	400(34.5)	256(22.0)		
Number of children	0	582	238(40.9)	235(40.4)	109(18.7)	9.052	0.060
	1	630	284(45.1)	216(34.3)	130(20.6)		
	≥2	417	169(40.5)	146(35.0)	102(24.5)		
Education	Graduate	57	19(33.3)	27(47.4)	11(19.3)	3.901	0.420
	Bachelor	1,496	643(43.0)	542(36.2)	311(20.8)		
	Diploma	77	30(39.0)	28(36.4)	19(24.7)		
Professional title	Nurse	205	86(42.0)	90(43.9)	29(14.1)	16.829	**0.010**
	Senior Nurse	539	228(42.3)	195(36.2)	116(21.5)		
	Charge Nurse	804	355(44.2)	277(34.5)	172(21.4)		
	Deputy Chief Nurse or above	82	23(28.0)	35(42.7)	24(29.3)		
Position level	N0	93	31(33.3)	42(45.2)	20(21.5)	42.006	**<0.001**
	N1	333	125(37.5)	144(43.2)	64(19.2)		
	N2	773	362(46.8)	251(32.5)	160(20.7)		
	N3	256	127(49.6)	85(33.2)	44(17.2)		
	N4	175	47(26.9)	75(42.9)	53(30.3)		
Years of experience	<4	359	139(38.7)	154(42.9)	66(18.4)	16.450	**0.012**
	4–7	214	87(40.7)	79(36.9)	48(22.4)		
	8–15	792	369(46.5)	264(33.3)	160(20.2)		
	>15	265	98(37.0)	100(37.7)	67(25.3)		
Monthly income (RMB)	≤5,000	517	243(47.0)	174(33.7)	100(19.3)	10.446	**0.034**
	5,001–9,000	898	375(41.8)	331(36.9)	192(21.4)		
	≥9,001	215	74(34.4)	92(42.8)	49(22.8)		
Overtime hours per day	0	109	34(31.2)	23(21.1)	52(47.7)	71.687	**<0.001**
	<1	844	324(38.4)	336(39.8)	184(21.8)		
	1–2	432	217(50.2)	151(35.0)	64(14.8)		
	>2	245	117(47.8)	87(35.5)	41(16.7)		
Night shifts per week	0	429	152(35.4)	164(38.2)	113(26.3)	29.901	**<0.001**
	1	411	154(37.5)	170(41.4)	87(21.2)		
	2	548	269(49.1)	181(33.0)	98(17.9)		
	≥3	242	117(48.3)	82(33.9)	43(17.8)		
Department system	Obstetrics–Gynecology and Pediatrics System	168	48(28.6)	63(37.5)	57(33.9)	81.704	**<0.001**
	Emergency and Critical Care System	210	102(48.6)	82(39.0)	26(12.4)		
	Outpatient and Medical Technology System	265	122(46.0)	78(29.4)	65(24.5)		
	Internal Medicine System	471	240(51.0)	161(34.2)	70(14.9)		
	Operating Room System	115	35(30.4)	53(46.1)	27(23.5)		
	Surgical System	314	127(40.4)	122(38.9)	65(20.7)		
	Oncology System	87	18(20.7)	38(43.7)	31(35.6)		
Specialty nurse	None	1,266	544(43.0)	455(35.9)	267(21.1)	6.685	0.351
	Hospital level	119	42(35.3)	47(39.5)	30(25.2)		
	Provincial level	159	72(45.3)	63(39.6)	24(15.1)		
	National level	86	34(39.5)	32(37.2)	20(23.3)		
Turnover intention	None	916	220(24.0)	396(43.2)	300(32.8)	346.371	**<0.001**
	Yes	623	396(63.6)	189(30.3)	38(6.1)		
	Very strong	91	76(83.5)	12(13.2)	3(3.3)		
Hospital level*	Secondary General Hospital	242	113(46.7)	87(36.0)	42(17.4)	2.965	0.227
	Tertiary General Hospital	1,388	579(41.7)	510(36.7)	299(21.5)		
Age (years)	Mean ± SD	1,630	33.11±6.44	32.54±6.68	33.48±6.96	2.430	0.088

*Note*: **Class 1**: Resource‐Deprived and High‐Stress Group; **Class 2**: Adaptive–Stable Group; **Class 3**: the High Resource–High Identity Group.

### 4.5 Factors Associated With Three Latent Profiles

Multinomial logistic regression was used to identify factors associated with membership in the three latent profiles: Resource‐Deprived and High‐Stress Group (C1), Adaptive–Stable Group (C2), and High Resource–High Identity Group (C3). The analysis revealed several significant predictors (all *p <* 0.05). Position level: Nurses with N2 positions were significantly more likely to belong to C1 compared with those with N4 positions (OR = 0.477, for C2 vs. C1; OR = 0.396, for C3 vs. C1). Monthly income: Nurses with a monthly income of ≤5,000 RMB were significantly more likely to belong to C1 compared with those earning ≥9,001 RMB (OR = 0.479, for C2 vs. C1; OR = 0.469, for C3 vs. C1). Department system: Nurses from the Outpatient were significantly more likely to belong to C1 compared with those from the Oncology System (OR = 0.316, for C2 vs. C1; OR = 0.242, for C3 vs. C1). Turnover intention: Nurses with no turnover intention were significantly less likely to belong to C1 compared with those with very strong turnover intention (OR = 8.658, for C2 vs. C1; OR = 20.999, for C3 vs. C1). For more detailed information, please refer to Table 3.

**TABLE 3:** Multinomial logistic regression for predicting external characteristics of three latent profiles.

Variables		Class 2 vs. Class 1 OR (95% CI)	*P*	Class 3 vs. Class 1 OR (95% CI)	*P*
Position level (N4 as ref)	N0	1.181 (0.435–3.203)	0.744	1.155 (0.331–4.034)	0.821
	N1	0.856 (0.393–1.862)	0.694	0.536 (0.211–1.364)	0.191
	N2	0.477 (0.273–0.833)	0.009	0.396 (0.205–0.763)	0.006
	N3	0.494 (0.281–0.867)	0.014	0.351 (0.178–0.690)	0.002
Marital status (Married as ref)	Unmarried	1.181 (0.798–1.747)	0.406	0.842 (0.510–1.389)	0.501
Years of experience (>15 years as ref)	<4	0.954 (0.424–2.143)	0.908	1.207 (0.446–3.267)	0.711
	4–7	1.239 (0.671–2.286)	0.493	1.553 (0.739–3.263)	0.246
	8–15	1.116 (0.733–1.700)	0.609	1.240 (0.746–2.060)	0.407
Professional title (Deputy Chief Nurse or above as ref)	Nurse	1.027 (0.423–2.494)	0.953	0.459 (0.148–1.417)	0.176
	Senior Nurse	1.015 (0.460–2.241)	0.970	1.173 (0.462–2.979)	0.738
	Charge Nurse	0.969 (0.481–1.954)	0.931	1.046 (0.463–2.362)	0.914
Monthly income (RMB, ≥9,001 as ref)	≤5,000	0.479 (0.308–0.747)	0.001	0.469 (0.269–0.817)	0.008
	5,001–9,000	0.700 (0.476–1.030)	0.070	0.744 (0.458–1.208)	0.232
Overtime hours per day (>2 as ref)	0	0.663 (0.328–1.340)	0.252	3.278 (1.556–6.909)	0.002
	<1	1.375 (0.907–2.085)	0.134	1.423 (0.822–2.465)	0.208
	1–2	1.129 (0.737–1.729)	0.576	0.903 (0.506–1.610)	0.729
Night shifts per week (≥3 as ref)	0	1.288 (0.819–2.026)	0.273	0.876 (0.493–1.555)	0.651
	1	1.215 (0.800–1.845)	0.360	0.727 (0.424–1.246)	0.246
	2	0.873 (0.598–1.275)	0.481	0.694 (0.420–1.144)	0.152
Department system (Oncology system as ref)	Obstetrics–Gynecology and Pediatrics System	0.966 (0.470–1.984)	0.924	1.147 (0.523–2.514)	0.733
	Emergency and Critical Care System	0.519 (0.262–1.031)	0.061	0.193 (0.085–0.436)	<0.001
	Outpatient and Medical Technology System	0.316 (0.159–0.628)	0.001	0.242 (0.113–0.516)	<0.001
	Internal Medicine System	0.477 (0.253–0.899)	0.022	0.289 (0.142–0.587)	<0.001
	Operating Room System	0.856 (0.380–1.929)	0.708	0.718 (0.281–1.833)	0.489
	Surgical System	0.627 (0.326–1.206)	0.162	0.383 (0.185–0.794)	0.010
Turnover intention (very strong as ref)	None	8.658 (4.497–16.668)	<0.001	20.999 (6.383–69.079)	<0.001
	Yes	2.330 (1.215–4.468)	0.011	1.642 (0.485–5.557)	0.425

*Note*: Class 1: Resource‐Deprived and High‐Stress Group; Class 2: Adaptive–Stable Group; Class 3: the High Resource–High Identity Group.

## 5 Discussion

Most existing research on nurses tends to focus on factors like organizational support, job satisfaction, or burnout at an overall level, with limited attention to the heterogeneity within the nursing population. This study, based on data from 1,630 clinical nurses across three general hospitals in two Chinese provinces, identified three latent subgroups characterized by distinct organizational–psychological features. These profiles revealed structural heterogeneity in nurses’ perceptions of PI, organizational resources, and subjective work experiences. Notably, the Resource‐Deprived and High‐Stress Group accounted for 42.0% of the sample, characterized by poor organizational support and unfavorable psychological status, characterizing them as a high‐priority population for managerial intervention. In contrast, only 21.0% of nurses were classified within the High Resource–High Identity Group, suggesting a relatively low proportion of highly adaptive and professionally engaged nurses. This imbalance highlights an urgent need to enhance organizational support systems that foster PI and psychological well‐being. The Resource‐Deprived and High‐Stress Group (C1) was significantly more likely to consist of nurses in junior positions (N2), those earning ≤5,000 RMB, working in high‐intensity departments like emergency care and outpatient settings, and those reporting a very strong intention to leave their jobs (*p* < 0.001). These findings offer valuable theoretical insights and practical implications for developing stratified intervention strategies and optimizing human resource allocation within clinical nursing management.

Building upon LPA results, this study identified three distinct subgroups of nurses characterized by structural differences in PI, organizational support, and work‐related experiences, revealing underlying heterogeneity in their organizational–psychological profiles. The Resource‐Deprived and High‐Stress Group represented the largest proportion of nurses, aligning with previous findings reported by Xue et al. (2025), revealing that this group predominantly comprised front‐line clinical nurses working in high‐intensity environments with relatively weak support systems. These nurses often experience a “high demand–low reward” dynamic, predisposing them to burnout and psychological exhaustion. A meta‐analysis conducted by Li et al. (2024), involving over 280,000 nurses across 32 countries, confirmed that burnout significantly increases the risk of adverse events such as medical errors and patient falls, ultimately compromising patient safety. It is therefore imperative that nurse managers implement focused interventions for this subgroup. For the Resource‐Deprived and High‐Stress Group, targeted strategies are essential. Managers should enhance communication and provide positive feedback, ensuring sufficient rest periods following night shifts and equitable financial compensation. Besides, structured, practical training programs that enhance clinical competence and professional confidence should be offered, avoiding fragmented and repetitive content. Lastly, fostering career development through learning opportunities can reinforce PI and improve occupational experiences. The Adaptive–Stable Group exhibited moderately high scores across most dimensions, indicative of strong professional adaptability and psychological resilience. As key contributors to the nursing workforce, these individuals often demonstrate robust organizational loyalty and task performance (Kida et al. 2023). To facilitate their continued progression, institutions should offer personalized career pathways, such as education, research, clinical expertise, or administration, based on individual preferences, while strengthening team culture and recognition mechanisms to facilitate their transition toward a high‐identity profile. The High Resource–High Identity Group achieved the highest scores across all domains, including PI, organizational support, and job satisfaction. As core members and potential leaders within the team, these nurses should be empowered with greater development opportunities and decision‐making authority. This includes providing access to advanced training, opportunities to lead research initiatives, and involvement in nursing management and specialty development, thereby fostering both personal advancement and collaborative excellence. In sum, implementing stratified management strategies tailored to the unique needs of each profile can enhance overall job satisfaction and organizational cohesion, advancing the precision and sustainability of nursing workforce development (Niskala et al. 2020).

Our findings suggest that specific sub‐dimensions, rather than the global constructs alone, play a critical role in shaping the latent organizational‐psychosocial profiles of nurses. For example, work stress—a core sub‐dimension of work‐related quality of life—stands out in the Resource‐Deprived and High‐Stress Group, consistent with evidence that high job stress strongly undermines nurses’ quality of life and increases burnout risk (Babapour et al. 2022). In contrast, sub‐dimensions of WSC, such as mutual trust, social support, and cohesion, seem more influential in the Adaptive–Stable and High Resource–High Identity groups, aligning with prior studies linking stronger social capital to better psychological well‐being, lower turnover intention, and higher job satisfaction (Xu et al. 2022). Moreover, PI sub‐aspects—such as professional social skills, professional self‐reflection, and professional cognitive assessment—in High Resource–High Identity groups show elevated levels, suggesting that robust PI components may buffer against stress and foster resilience and engagement. This matches prior findings that high PI correlates with better work adjustment and retention among nurses (Xu et al. 2024). Thus, the interplay of stress, social capital, and identity sub‐dimensions appears to underpin the distinct nurse profiles observed in this study. Recognizing which sub‐dimensions drive vulnerability vs. stability can guide more targeted interventions — e.g., stress‐reduction strategies for C1; social‐capital building for C2; identity reinforcement for C3—thereby improving nurse well‐being and workforce sustainability.

This study identified positional seniority as a significant factor influencing nurses' classification into the Resource‐Deprived and High‐Stress Group. Nurses in N2 and N3 positions, with moderate experience but lacking the full maturity of N4 nurses, often serve as the ‘middle force’ in their departments. This role requires them to take on heavier workloads, which increases their stress (Tatala et al. 2025). Besides, their underdeveloped professional social networks contribute to a weaker sense of organizational support (Jarden et al. 2021). To address these challenges, nursing managers should adopt a strategic approach to task allocation to balance workload with opportunities for growth and psychological resilience. A mentorship system can be implemented, where experienced nurses provide guidance on professional skills, communication, and career planning (Jarden et al. 2021). Training initiatives should prioritize high‐quality, practical, and structured content, such as case discussions and risk management, to enhance professional competence. Furthermore, establishing transparent and efficient communication channels ensures the timely dissemination of policies, as well as open feedback and grievance mechanisms, thereby increasing nurses’ engagement and psychological safety. Performance management systems should incorporate clear, publicly accessible evaluation criteria, integrating multidimensional metrics (e.g., nursing safety and patient satisfaction) to improve motivation. A systematic support framework can help early‐career nurses develop, improve retention, and strengthen the overall stability and resilience of the nursing workforce.

Research indicates that unit type significantly influences nurses’ occupational stress levels. Nurses working in high‐risk and high‐intensity departments, such as outpatient and diagnostic units, and the Internal Medicine System, are more likely to experience sustained work‐related stress due to complex patient conditions, fast‐paced workflows, and staffing shortages (Maghsoud et al. 2022; Bolado et al. 2024). To address this, hospital administrators should adopt multifaceted strategies to optimize resource allocation in these departments, including strictly enforcing national nurse‐to‐bed ratio standards, ensuring scientific staffing practices, and increasing compensation to enhance job attractiveness and perceived fairness. Preferential treatment in performance evaluations, awards, and professional promotion should also be considered to boost motivation. Guaranteeing annual leave entitlements can alleviate physical and mental fatigue. Besides, implementing flexible scheduling models can help achieve a better work–life balance, promoting both rest and productivity, thereby improving job stability and work‐related well‐being. It is also recommended to establish a “nurse resource pool” as a flexible staffing reserve. Based on real‐time bed occupancy rates, resource nurses can be dispatched to support high‐demand units, relieving temporary staffing pressures and enhancing the resilience and responsiveness of nursing workforce deployment.

Moreover, our findings confirmed that monthly income is a significant factor influencing nurses’ likelihood of belonging to the high‐stress group. Indeed, lower income often creates a perception of effort–reward imbalance, thereby undermining a nurse's motivation (Braun et al. 2024). Studies have highlighted that increasing compensation is a key strategy for retaining nurses with turnover intentions (Pressley and Garside 2023). Hospital administrators should therefore optimize the performance‐based compensation system through evidence‐based approaches, specifically by ensuring baseline compensation by setting appropriate performance levels according to educational background, years of experience, and professional titles, thereby promoting fairness and professional respect. Besides, incentive‐based pay should be aligned with job‐related risks, workload intensity, and responsibilities, thereby recognizing nurses’ professional value and strengthening their job satisfaction and sense of achievement. Over the years, performance‐based payment systems matching nursing workload and task complexity have been explored in several countries. For example, models piloted in Australia have linked compensation with nursing‐sensitive indicators and patient dependency levels (Duffield et al. 2010). Overall, institutional recognition of nurses’ labor value may foster stronger PI and a greater sense of accomplishment, while reducing the risk of turnover.

This study identified turnover intention as a significant predictor of nurse inclusion in the “Resource‐Deprived and High‐Stress Group.” Turnover intention refers to an individual's subjective willingness to voluntarily leave their current position in the foreseeable future and is often a cumulative reflection of burnout, organizational disengagement, and declining job satisfaction (Hu et al. 2022). Recent studies have shown a steady rise in turnover intention among nurses, particularly in clinical environments characterized by high stress and inadequate organizational support (Kim and Kim 2021; Namin et al. 2021). Beyond signaling potential workforce loss, turnover intention may also lead to disengaged work attitudes, avoidance of responsibilities, reduced team efficiency, and ultimately jeopardize patient safety and clinical outcomes (Poku et al. 2025). Nurse managers should regularly collect multidimensional data related to nurses’ occupational status and develop data‐driven mechanisms to identify and intervene with at‐risk individuals at an early stage. For high‐risk nurses, structured interviews, optimized task allocation, and personalized career counseling can be implemented to help clarify development pathways and enhance their sense of control and achievement. Besides, establishing a systematic psychological support system, such as stress‐reduction consultations and group counseling, may help alleviate emotional exhaustion induced by prolonged occupational stress.

## 6 Limitations

This study lies in its large‐scale, multicenter survey involving 1,630 nurses. Guided by occupational health psychology and social capital theory, the study explored the impact of WRQoL, WSC, and PI on the characteristics of the nursing workforce. By uncovering the multidimensional profiles of nurses, this study provides theoretical foundations and empirical support for developing targeted management strategies to enhance nurses' well‐being and work performance. However, several limitations should be acknowledged. First, the cross‐sectional design limits the ability to infer causal relationships among variables. Future longitudinal studies are needed to examine the dynamic changes in nurses’ psychosocial–organizational characteristics and their long‐term effects. However, even longitudinal studies cannot guarantee precise causal relationships due to limitations such as difficulty in controlling confounding variables, potential reverse causality, and uncertainties in effect timing. Second, the sample was drawn from three general hospitals in two provinces of China using convenience sampling, which may limit the geographical and institutional representativeness of the findings. Therefore, caution should be exercised when generalizing the results to other regions or healthcare settings. Finally, the data were based on self‐reported measures, which may be subject to social desirability bias and CMV. Future studies should consider incorporating multisource data, such as supervisor evaluations and patient feedback, to enhance the accuracy and robustness of the measurements.

## 7 Conclusion

This study identified three latent organizational–psychosocial profiles among clinical nurses, namely, the Resource‐Deprived and High‐Stress Group, the Adaptive–Stable Group, and the High‐Resource–High‐Identity Group, based on WSC, PI, and WRQoL. Our findings revealed significant heterogeneity in perceived organizational support and subjective work experience among nurses, with the Resource‐Deprived and High‐Stress Group accounting for as high as 42.0% of the sample, indicating a widespread prevalence of psychosocial vulnerability within the nursing workforce. Multinomial logistic regression identified position level, clinical specialty, monthly income, and turnover intention as key predictors of membership in the high‐stress group. These results underscore the impact of structural factors—such as position level, department, and income—on nurses’ work‐related well‐being and PI. By adopting a person‐centered approach, this study uncovers the hidden stratification within nurses’ work‐related well‐being and provides empirical evidence for developing targeted workforce management strategies. Moving forward, nurse managers should implement differentiated support policies tailored to distinct nurse profiles, optimize resource allocation and career development pathways, and enhance PI and job satisfaction to strengthen workforce sustainability and improve care quality.

## 8 Implications for Nursing and Health Policy

This study reveals substantial variation in psychological and organizational experiences across distinct nurse profiles, underscoring the need to shift nursing management from a “one‐size‐fits‐all” model toward a stratified and differentiated approach to improve intervention efficacy. Nurse managers should implement a tiered management framework that enables precise allocation of support and resources. Priority interventions should target nurses in the Resource‐Deprived and High‐Stress Group, including optimizing shift schedules, reducing workload, and providing robust psychological support to alleviate physical and emotional strain. Simultaneously, emotional support and value‐based feedback should be strengthened to promote a sense of belonging and prevent burnout. For junior nurses in particular, mentorship programs and collaborative practice models can enhance clinical competence and team integration. Indeed, nurses in the Adaptive–Stable Group should be offered well‐defined career development pathways, including specialty training, research participation, innovation opportunities, and role rotation, to unlock their full potential and reduce the risk of stagnation or attrition. For those in the High Resource–High Identity Group, efforts should be made to foster their leadership potential through teaching, managerial involvement, and decision‐making opportunities, thereby enhancing their sense of professional fulfillment and cultivating intrinsic team motivation. Hierarchical position and department assignment can serve as early warning indicators for workforce risk. It is recommended that hospitals establish a dynamic system for assessing nurses’ professional status to identify both at‐risk individuals and high‐potential talent, thereby providing data‐driven support for nursing human resource management.

## Author Contributions

Study design: WLL, XMW, YW, and WJZ. Data collection: LZ, XXC, and WJZ. Data analysis: DNX and NX. Study supervision: WLL. Manuscript writing: WJZ and LZ. Critical revisions for important intellectual content: YW, WLL, and WJZ.

## Conflicts of Interest

The authors declare no conflicts of interest.

## Ethical Approval

Participation was voluntary, and data were collected anonymously. Written informed consent was not required for this online survey. Participants were informed that they could withdraw anytime, and the final questionnaire was not shared with nursing management. The study was approved by the ethics committee of Shanxi Bethune Hospital (No. YXLL‐2023‐229). No patients or members of the public were involved in the design, conduct, or reporting of this research.

## Funding

The authors have nothing to report.

## Supporting information




**Supporting Table 1**: Internal Consistency of Each Scale Dimension (Cronbach's α).

**Supporting Table 2**: Correlation Analysis among Dimensions of Workplace Social Capital, Professional Identity, and Work‐Related Quality of Life.

**Supporting Table 3**: Common Method Variance (CMV) Test Results.

## Data Availability

The data supporting the findings of this study are not publicly available due to privacy and ethical restrictions.

## References

[inr70192-bib-0001] Akter, N. , M. Akter , and S. Turale . 2019. “Barriers to Quality of Work Life Among Bangladeshi Nurses: A Qualitative Study.” International Nursing Review 66, no. 3: 396–403. 10.1111/inr.12540. 31393005

[inr70192-bib-0002] Al Mutair, A. , M. I. Al Bazroun , E. M. Almusalami , et al. 2022. “Quality of Nursing Work Life Among Nurses in Saudi Arabia: A Descriptive Cross‐Sectional Study.” Nursing Reports 12, no. 4: 1014–1022. 10.3390/nursrep12040097. 36548170 PMC9783332

[inr70192-bib-0003] Andrew, N. 2012. “Professional Identity in Nursing: Are We There Yet?” Nurse Education Today 32, no. 8: 846–849. 10.1016/j.nedt.2012.03.014. 22531469

[inr70192-bib-0004] Babapour, A. , N. Gahassab‐Mozaffari , and A. Fathnezhad‐Kazemi . 2022. “Nurses' Job Stress and Its Impact on Quality of Life and Caring Behaviors: A Cross‐Sectional Study.” BMC Nursing 21, no. 1: 75. 10.1186/s12912-022-00852-y. 35361204 PMC8968092

[inr70192-bib-0005] Bolado, G. N. , B. A. Ataro , C. K. Gadabo , A. S. Ayana , T. E. Kebamo , and W. M. Minuta . 2024. “Stress Level and Associated Factors Among Nurses Working in the Critical Care Unit and Emergency Rooms at Comprehensive Specialized Hospitals in Southern Ethiopia, 2023: Explanatory Sequential Mixed‐Method Study.” BMC Nursing 23, no. 1: 341. 10.1186/s12912-024-02004-w. 38773519 PMC11106981

[inr70192-bib-0006] Braun, J. , S. Darius , and I. Böckelmann . 2024. “The Correlation between Effort–Reward Imbalance at Work and the Risk of Burnout among Nursing Staff Working in an Emergency Department—A Pilot Study.” Healthcare (Basel) 12, no. 22: 2249. 10.3390/healthcare12222249. 39595447 PMC11593360

[inr70192-bib-0007] Duffield, C. , M. Roche , D. Diers , C. Catling‐Paull , and N. Blay . 2010. “Staffing, Skill Mix and the Model of Care.” Journal of Clinical Nursing 19, no. 15–16: 2242–2251. 10.1111/j.1365-2702.2010.03225.x. 20659202

[inr70192-bib-0008] Firouzbakht, M. , A. Tirgar , T. Oksanen , et al. 2018. “Workplace Social Capital and Mental Health: A Cross‐Sectional Study among Iranian Workers.” BMC Public Health 18, no. 1: 794. 10.1186/s12889-018-5659-3. 29940919 PMC6019288

[inr70192-bib-0009] Fitzgerald, A. 2020. “Professional Identity: A Concept Analysis.” Nursing Forum 55, no. 3: 447–472. 10.1111/nuf.12450. 32249453

[inr70192-bib-0010] Hu, H. , C. Wang , Y. Lan , and X. Wu . 2022. “Nurses' Turnover Intention, Hope and Career Identity: The Mediating Role of Job Satisfaction.” BMC Nursing 21, no. 1: 43. 10.1186/s12912-022-00821-5. 35144604 PMC8830989

[inr70192-bib-0011] International Council of Nurses . 2025. “ICN Launches New Report and Survey Warning of Deepening Global Nursing Crisis, Offering Solutions.” https://www.icn.ch/news/icn‐launches‐new‐report‐and‐survey‐warning‐deepening‐global‐nursing‐crisis‐offering‐solutions.

[inr70192-bib-0012] Jarden, R. J. , A. Jarden , T. J. Weiland , G. Taylor , N. Brockenshire , and M. Gerdtz . 2021. “Registered Nurses' Experiences of Psychological Well‐Being and Ill‐Being in Their First Year of Practice: A Qualitative Meta‐Synthesis.” Journal of Advanced Nursing 77, no. 3: 1172–1187. 10.1111/jan.14667. 33314252

[inr70192-bib-0013] Johnson, M. , L. Cowin , I. Wilson , and H. Young . 2012. “Professional Identity and Nursing: Contemporary Theoretical Developments and Future Research Challenges.” International Nursing Review 59, no. 4: 562–569. 10.1111/j.1466-7657.2012.01013.x. 23134142

[inr70192-bib-0014] Kida, R. , Y. Yumoto , and Y. Ogata . 2023. “Workplace Social Capital Mediates the Relationship Between Authentic Leadership and Three Dimensions of Organizational Commitment of Hospital Nurses: A Cross‐Sectional Study.” Japanese Journal of Nursing Science 20, no. 3: e12526. 10.1111/jjns.12526. 36752048

[inr70192-bib-0015] Kim, H. , and E. Kim . 2021. “A Meta‐Analysis on Predictors of Turnover Intention of Hospital Nurses in South Korea (2000–2020).” Nursing Open 8, no. 5: 2406–2418. 10.1002/nop2.872. 33826252 PMC8363357

[inr70192-bib-0016] Kouvonen, A. , M. Kivimäki , J. Vahtera , et al. 2006. “Psychometric Evaluation of a Short Measure of Social Capital at Work.” BMC Public Health 6: 251. 10.1186/1471-2458-6-251. 17038200 PMC1618843

[inr70192-bib-0017] Landis, T. , B. Severtsen , M. Shaw , and C. Holliday . 2020. “Professional Identity and Hospital‐Based Registered Nurses: A Phenomenological Study.” Nursing Forum 55, no. 3: 389–394. 10.1111/nuf.12440. 32096218

[inr70192-bib-0018] Li, L. Z. , P. Yang , S. J. Singer , J. Pfeffer , M. B. Mathur , and T. Shanafelt . 2024. “Nurse Burnout and Patient Safety, Satisfaction, and Quality of Care: A Systematic Review and Meta‐Analysis.” JAMA Network Open 7, no. 11: e2443059. 10.1001/jamanetworkopen.2024.43059. 39499515 PMC11539016

[inr70192-bib-0019] Li, P. , Y. Wang , and M. Zhang . 2022. “Translation and Validation of the Work‐Related Quality of Life Scale (WRQoLS‐2) in a Nursing Cohort.” Contemporary Nurse 58, no. 5‐6: 435–445. 10.1080/10376178.2022.2147849. 36377362

[inr70192-bib-0020] Liu, L. 2009. “The Level of Professional Identity Among Nurses and Its Relationship With Job Stress and Burnout.” Master's thesis, Second Military Medical University, Shanghai, China.

[inr70192-bib-0021] Liukkonen, V. , P. Virtanen , M. Kivimäki , J. Pentti , and J. Vahtera . 2004. “Social Capital in Working Life and the Health of Employees.” Social Science & Medicine 59, no. 12: 2447–2458. 10.1016/j.socscimed.2004.04.013. 15474200

[inr70192-bib-0022] Maghsoud, F. , M. Rezaei , F. Asgarian , and M. Rassouli . 2022. “Workload and Quality of Nursing Care: The Mediating Role of Implicit Rationing of Nursing Care, Job Satisfaction and Emotional Exhaustion by Using Structural Equations Modeling Approach.” BMC Nursing 21, no. 1: 273. 10.1186/s12912-022-01055-1. 36209155 PMC9548180

[inr70192-bib-0023] Mao, A. , S. Lu , Y. Lin , and M. He . 2021. “A Scoping Review on the Influencing Factors and Development Process of Professional Identity Among Nursing Students and Nurses.” Journal of Professional Nursing 37, no. 2: 391–398. 10.1016/j.profnurs.2020.04.018. 33867096

[inr70192-bib-0024] Melnyk, B. M. , L. Gallagher‐Ford , C. Zellefrow , et al. 2018. “The First U.S. Study on Nurses' Evidence‐Based Practice Competencies Indicates Major Deficits That Threaten Healthcare Quality, Safety, and Patient Outcomes.” Worldviews on Evidence‐Based Nursing 15, no. 1: 16–25. 10.1111/wvn.12269. 29278664

[inr70192-bib-0025] Namin, B. , T. Øgaard , and J. Røislien . 2021. “Workplace Incivility and Turnover Intention in Organizations: A Meta‐Analytic Review.” International Journal of Environmental Research and Public Health 19, no. 1: 25. 10.3390/ijerph19010025. 35010292 PMC8751201

[inr70192-bib-0026] Niskala, J. , O. Kanste , M. Tomietto , et al. 2020. “Interventions to Improve Nurses' Job Satisfaction: A Systematic Review and Meta‐Analysis.” Journal of Advanced Nursing 76, no. 7: 1498–1508. 10.1111/jan.14342. 32128864

[inr70192-bib-0027] Pittman, J. , A. Cohee , S. Storey , et al. 2019. “A Multisite Health System Survey to Assess Organizational Context to Support Evidence‐Based Practice.” Worldviews on Evidence‐Based Nursing 16, no. 4: 271–280. 10.1111/wvn.12375. 31231947

[inr70192-bib-0028] Podsakoff, P. , S. MacKenzie , J. Lee , and N. Podsakoff . 2003. “Common Method Biases in Behavioral Research: A Critical Review of the Literature and Recommended Remedies.” Journal of Applied Psychology 88, no. 5: 879–903. 10.1037/0021-9010.88.5.879. 14516251

[inr70192-bib-0029] Poku, C. A. , J. Bayuo , V. A. Agyare , N. K. Sarkodie , and V. Bam . 2025. “Work Engagement, Resilience and Turnover Intentions Among Nurses: A Mediation Analysis.” BMC Health Services Research 25, no. 1: 71. 10.1186/s12913-025-12242-6. 39806365 PMC11730472

[inr70192-bib-0030] Pressley, C. , and J. Garside . 2023. “Safeguarding the Retention of Nurses: A Systematic Review on Determinants of Nurse's Intentions to Stay.” Nursing Open 10, no. 5: 2842–2858. 10.1002/nop2.1588. 36646646 PMC10077373

[inr70192-bib-0031] Quick, J. , and L. Tetrick . 1993. Handbook of Occupational Health Psychology. American Psychological Association. https://www.apa.org/pubs/books/handbook‐occupational‐health‐psychology.

[inr70192-bib-0032] Rashid, I. , and F. Amin . 2024. “Mediating Role of Quality of Work Life Between Work‐Related Social Capital and Life Satisfaction Among Health Professionals.” Arab Gulf Journal of Scientific Research 42, no. 4: 1700–1715. 10.1234/arabgulf.2023.1715.

[inr70192-bib-0033] Silarova, B. , N. Brookes , S. Palmer , A. Towers , and S. Hussein . 2022. “Understanding and Measuring the Work‐Related Quality of Life Among Those Working in Adult Social Care: A Scoping Review.” Health & Social Care in the Community 30, no. 5: 1637–1664. 10.1111/hsc.13718. 35066964 PMC9543435

[inr70192-bib-0034] Spurk, D. , A. Hirschi , M. Wang , D. Valero , and S. Kauffeld . 2020. “Latent Profile Analysis: A Review and “How to” Guide of Its Application Within Vocational Behavior Research.” Journal of Vocational Behavior 120: 103445. 10.1016/j.jvb.2020.103445.

[inr70192-bib-0035] Tatala, M. , M. Wojtasiński , K. Janowski , and P. Tużnik . 2025. “Perceived Stress and Burnout in Nurses: The Moderating Role of Age and Network Analysis Perspective.” Annals of Agricultural and Environmental Medicine 32, no. 1: 85–97. 10.26444/aaem/191048. 40159740

[inr70192-bib-0036] Van Laar, D. , J. Edwards , and S. Easton . 2007. “The Work‐Related Quality of Life Scale for Healthcare Workers.” Journal of Advanced Nursing 60, no. 3: 325–333. 10.1111/j.1365-2648.2007.04409.x. 17908128

[inr70192-bib-0037] von Elm, E. , D. G. Altman , M. Egger , S. J. Pocock , P. C. Gøtzsche , and J. P. Vandenbroucke . 2007. “The Strengthening the Reporting of Observational Studies in Epidemiology (STROBE) Statement: Guidelines for Reporting Observational Studies.” Annals of Internal Medicine 147, no. 8: 573–577. 10.7326/0003-4819-147-8-200710160-00010. 17938396

[inr70192-bib-0038] Xu, J. , M. Cao , Q. Gao , Y. Lu , and A. T. Stark . 2024. “Nurses' Workplace Social Capital and Sustainable Development: An Integrative Review of Empirical Studies.” Journal of Nursing Management 2024: 8362035. 10.1155/2024/8362035. 40224862 PMC11918928

[inr70192-bib-0039] Xu, J. , W. Kunaviktikul , T. Akkadechanunt , A. Nantsupawat , and S. Turale . 2021. “Factors Influencing Workplace Social Capital Among Registered Nurses in China.” International Nursing Review 68, no. 3: 372–379. 10.1111/inr.12666. 33639024

[inr70192-bib-0040] Xu, J. , and A. Stark . 2021. “A Conceptual Model of Nurses' Workplace Social Capital: A Theory Synthesis.” BMC Nursing 20, no. 1: 148. 10.1186/s12912-021-00660-w. 34404398 PMC8369697

[inr70192-bib-0041] Xu, J. , A. T. Stark , B. Ying , Z. Lian , Y. Huang , and R. Chen . 2022. “Nurses' Workplace Social Capital and the Influence of Transformational Leadership: A Theoretical Perspective.” Frontiers in Public Health 10: 855278. 10.3389/fpubh.2022.855278. 35769783 PMC9234161

[inr70192-bib-0042] Xue, F. , J. Liu , T. Zhou , et al. 2025. “The Relationship Between Burnout, Sense of Coherence and Job Safety Attitudes Among Nurses After Coronavirus Disease 2019 in China: A Cross‐sectional Survey.” Frontiers in Public Health 13: 1516744. 10.3389/fpubh.2025.1516744. 40046128 PMC11879944

[inr70192-bib-0043] Zhang, N. , L. Zhang , and Z. Liang . 2014. “Revision and Evaluation of the Workplace Social Capital Scale.” Chinese Journal of Health Statistics 31, no. 4: 456–458.

