# Supplementary material for: Workplace Social Capital, Professional Identity, and Work‐Related Quality of Life Among Nurses: A Latent Profile Analysis
Source: Int Nurs Rev. 2026 Jun 22;73(2):e70192. doi: 10.1111/inr.70192 (PMC13287914; doi:10.1111/inr.70192)
Supplement: Supplementary file 1 — Supporting Table 1: Internal Consistency of Each Scale Dimension (Cronbach's α). [file INR-73-0-s001.docx]

# Supplementary Table S1.

# Internal Consistency of Each Scale Dimension (Cronbach’s α)

| **Scale** | **Dimension** | **Item Code** | **Cronbach’s α** |
| --- | --- | --- | --- |
| Workplace Social Capital | **bonding social capital** | V1 | 0.95 |
|  | **non-bonding social capital** | V2 | 0.95 |
| Professional Identity | Cognitive Evaluation | V3 | 0.94 |
|  | Social Skills | V4 | 0.94 |
|  | Social Support | V5 | 0.94 |
|  | Frustration Coping | V6 | 0.94 |
|  | Self-Reflection | V7 | 0.94 |
|  | Self-Reflection | V7 | 0.94 |
| Work-Related Quality of Life | Control at Work | V8 | 0.94 |
|  | General Wellbeing | V9 | 0.94 |
|  | Work Stress | V10 | 0.97 |
|  | Working Conditions | V11 | 0.94 |
|  | Work-Family Balance | V12 | 0.94 |
|  | Work Evaluation | V13 | 0.94 |
|  | Career Satisfaction | V14 | 0.95 |
