# Supplementary material for: Workplace Social Capital, Professional Identity, and Work‐Related Quality of Life Among Nurses: A Latent Profile Analysis
Source: Int Nurs Rev. 2026 Jun 22;73(2):e70192. doi: 10.1111/inr.70192 (PMC13287914; doi:10.1111/inr.70192)
Supplement: Supplementary file 2 — Supporting Table 2: Correlation Analysis among Dimensions of Workplace Social Capital, Professional Identity, and Work‐Related Quality of Life. [file INR-73-0-s002.docx]

# Supplementary Table S2.

# Correlation Analysis among Dimensions of Workplace Social Capital, Professional Identity, and Work-Related Quality of Life




Note:

V1–V2 = Workplace Social Capital;

V3–V7 = Professional Identity;

V8–V14 = Work-Related Quality of Life.

All correlations are significant at *p < 0.001.*
