# Supplementary material for: Workplace Social Capital, Professional Identity, and Work‐Related Quality of Life Among Nurses: A Latent Profile Analysis
Source: Int Nurs Rev. 2026 Jun 22;73(2):e70192. doi: 10.1111/inr.70192 (PMC13287914; doi:10.1111/inr.70192)
Supplement: Supplementary file 3 — Supporting Table 3: Common Method Variance (CMV) Test Results. [file INR-73-0-s003.docx]

# Supplementary Table S3. **Common Method Variance (CMV) Test Results**

Since data in this study were collected through self-reported questionnaires, there is a potential risk of Common Method Variance (CMV).

**1 Harman’s Single-Factor Test**

To assess the potential impact of CMV, we conducted Harman’s Single-Factor Test by including all observed variables (33 items from the WRQoL scale, 8 items from the WSC scale, and 30 items from the PI scale, totaling 71 items) in an exploratory factor analysis (EFA). Unrotated principal component analysis (PCA) was used to extract the common factors.

The results showed that the first common factor explained 44.98% of the variance, which is below the 50% threshold. This indicates that CMV is not a dominant factor in our data, suggesting that CMV did not significantly affect the results.

**2 Confirmatory Factor Analysis (CFA) Model Comparison**

To further validate the presence of CMV, we compared the fit of a three-factor model (baseline model) and a single-factor model (CMV hypothesis model) using Confirmatory Factor Analysis (CFA). Both models were analyzed using Mplus 5.1 software with robust maximum likelihood estimation (MLR) to account for non-normal data.

The following fit indices were used to assess model fit: CFI (Comparative Fit Index), TLI (Tucker-Lewis Index), RMSEA (Root Mean Square Error of Approximation), SRMR (Standardized Root Mean Square Residual)

**Table 1: Comparison of Model Fit Indices for Competing Models**

|  | CFI | TLI | RMSEA | SRMR |
| --- | --- | --- | --- | --- |
| model1 | 0.830 | 0.825 | 0.062 | 0.053 |
| model2 | 0.687 | 0.678 | 0.085 | 0.077 |

Model 1 (Three-Factor Model) showed acceptable fit indices (CFI = 0.83, TLI = 0.825, RMSEA = 0.062, SRMR = 0.053), indicating good fit for the proposed theoretical model.

Model 2 (Single-Factor Model), hypothesizing that all items load onto a single common factor, showed poor fit (CFI = 0.687, TLI = 0.678, RMSEA = 0.085, SRMR = 0.077), with all indices failing to meet acceptable standards. This indicates that the true data structure is three-factor, not a false single-factor structure induced by CMV.

**3 Conclusion**

Based on the results from both Harman’s Single-Factor Test and CFA model comparison, we conclude that CMV is not a significant issue in the data. The three-factor model provides a better fit and supports the validity of the constructs, confirming that CMV did not substantially affect the relationships among the variables in this study. Therefore, no further CMV correction is required.
